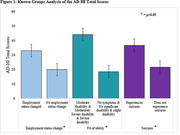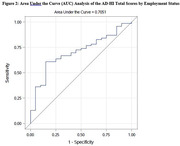# The Alzheimer’s Disease‐Health Index (AD‐HI): a novel, disease‐specific, patient‐reported outcome measure for use in Alzheimer’s disease clinical trials and patient monitoring

**DOI:** 10.1002/alz.085604

**Published:** 2025-01-09

**Authors:** Charlotte Engebrecht

**Affiliations:** ^1^ Center for Health + Technology, University of Rochester Medical Center, Rochester, NY USA

## Abstract

**Background:**

To bolster clinical trial infrastructure, there is a need to develop novel, valid, and reliable patient‐reported outcome (PRO) measures capable of tracking clinically‐relevant changes in Alzheimer’s disease (AD), Mild Cognitive Impairment (MCI) and dementia over time. This research describes the development and validation of the Alzheimer’s Disease‐Health Index (AD‐HI) as a tool to measure how patients feel and function in response to therapeutic intervention.

**Method:**

We previously conducted semi‐structured qualitative interviews and a national cross‐sectional study with individuals with AD, MCI and dementia to ascertain the most prevalent and impactful symptoms identified by the participants. This data was used to generate the first version of the AD‐HI. We then conducted beta testing of the AD‐HI with individuals with AD and MCI to determine the instrument’s comprehensibility, ease of use, and applicability to the AD and MCI populations. Following participant feedback, we further modified the instrument and conducted test‐retest reliability, known groups validity (**Figure 1**), area under the curve (AUC) analysis (**Figure 2**), and factor analysis to validate the AD‐HI.

**Result:**

Fifteen individuals with AD and MCI participated in the qualitative interviews and 104 individuals participated in the cross‐sectional study. We conducted beta testing with an additional 15 individuals with AD and MCI who stated that the AD‐HI was easy to use, clear, and highly relevant. Twenty‐three individuals with AD, MCI and dementia participated in test‐retest analysis. Known groups analysis demonstrated that the AD‐HI was able to differentiate between subgroups with varying levels of disease burden (based on employment status, disability status and those experiencing seizures).

**Conclusion:**

The AD‐HI is a valid and reliable regulatory‐grade instrument capable of measuring multifaceted disease burden in AD, MCI and dementia. The development, optimization and validation of the AD‐HI provides researchers and clinicians with a disease‐specific PRO for use in therapeutic trials and to potentially support FDA drug‐labeling claims.